# Comparison of SS-EPI DWI and one-minute TGSE-BLADE DWI for diagnosis of acute infarction

**DOI:** 10.1038/s41598-025-90413-5

**Published:** 2025-02-22

**Authors:** Sachi Okuchi, Yasutaka Fushimi, Akihiko Sakata, Sayo Otani, Satoshi Nakajima, Takakuni Maki, Masahiro Tanji, Noritaka Sano, Satoshi Ikeda, Shuichi Ito, Yuta Urushibata, Kun Zhou, Yoshiki Arakawa, Yuji Nakamoto

**Affiliations:** 1 Department of Diagnostic Imaging and Nuclear Medicine, Graduate School of Medicine, Kyoto University, 54 Shogoin Kawahara-cho, Sakyo-ku Kyoto, 606-8507 Japan; 2 Department of Neurology, Graduate School of Medicine, Kyoto University, Kyoto, Japan; 3 Department of Neurosurgery, Graduate School of Medicine, Kyoto University, Kyoto, Japan; 4 Siemens Healthcare K.K, Tokyo, Japan; 5 Siemens Shenzhen Magnetic Resonance Ltd, Shenzhen, China

**Keywords:** Diffusion-weighted imaging, Single-shot echo-planar imaging, TGSE-BLADE, Acute cerebral infarction, Stroke, Nervous system, Cerebrovascular disorders, Stroke

## Abstract

The efficacy of 2D turbo gradient- and spin-echo diffusion-weighted imaging with non-Cartesian BLADE trajectory (TGSE-BLADE DWI) has not been well studied for acute stroke due to its long acquisition time. This study was performed to compare distortion, artifacts and image quality between single-shot echo planar imaging (SS-EPI) DWI and TGSE-BLADE DWI with acquisition time reduced to 1 min by simultaneous multi-slice (SMS) imaging, and to evaluate the diagnostic performance of TGSE-BLADE DWI for acute infarctions. Total 104 patients with a past history of stroke or symptoms suspicious for acute infarction or who had undergone surgery for brain tumor within two days were prospectively enrolled. Ten lesions in 9 patients were diagnosed as acute or subacute infarction and were detectable only in TGSE-BLADE DWI but not in SS-EPI DWI. Scores for geometric distortion, susceptibility artifacts, overall image quality, lesion conspicuity and diagnostic confidence were lower for SS-EPI DWI than TGSE-BLADE DWI (*p* ≤ .001). Distortion was significantly worse in SS-EPI DWI than TGSE-BLADE DWI (*p* < .001). SNR of centrum semiovale was significantly higher in SS-EPI DWI than TGSE-BLADE DWI (*p* < .001). One-minute TGSE-BLADE DWI showed better image quality than SS-EPI DWI in terms of distortion and artifacts, and higher diagnostic performance for acute infarctions.

**Supplementary Information:**

The online version contains supplementary material available at 10.1038/s41598-025-90413-5.

## Introduction

Diffusion-weighted magnetic resonance imaging (DWI) is the most important MR sequence for diagnosing acute stroke^1,2^. Single-shot echo-planar imaging (SS-EPI) is the most widely used DWI technique; however, EPI-based DWI techniques are prone to susceptibility artifacts where the magnetic field is inhomogeneous, such as near air–bone interfaces^3^. In contrast, two-dimensional (2D) turbo gradient- and spin-echo diffusion-weighted imaging with non-Cartesian BLADE trajectory (TGSE-BLADE DWI) is insensitive to B_0_-related artifacts, and thus has reduced geometric distortion and susceptibility artifacts^4^. Although several studies have reported the clinical usefulness of TGSE-BLADE DWI for cholesteatomas, orbital tumors, cerebellopontine angle tumors, sinonasal lesions, and aneurysm clips^5–10^, none has evaluated its use for acute stroke. TGSE-BLADE DWI features a multi-blade k-space filling strategy that has a shorter acquisition time compared to PROPELLER DWI, which is based on a turbo spin-echo sequence with non-Cartesian BLADE trajectory^11–13^. However, the acquisition time for TGSE-BLADE DWI has been reported to be as long as 4–5 min^4–10^, which has prevented its clinical application.

To overcome this shortcoming, we used a slice acceleration technique termed simultaneous multi-slice (SMS) imaging in TGSE-BLADE DWI. SMS has been incorporated into both TSE and EPI sequences, and applied to most anatomical regions^14–17^. As SMS offers a substantial acceleration in data acquisition according to the number of slices excited simultaneously, it has emerged as a significant imaging technique^18^. In contrast to in-plane parallel imaging, SMS incurs only a minimal intrinsic signal-to-noise ratio penalty, allowing for full acceleration while maintaining a fixed echo time^19^. In addition, some SMS implementations have the potential to decrease radiofrequency (RF) power deposition^19^.

MRI is a crucial diagnostic tool for cerebral infarction that enables early detection and prompt formulation and initiation of treatment, which are correlated with enhanced patient prognosis^20,21^. Therefore, reduction of scan time is clinically important for increasing the efficacy of patient care. We achieved a reduction in TGSE-BLADE DWI acquisition time to 1 min by employing SMS. The aim of this study was to compare distortion, artifacts, and image quality between SS-EPI DWI and TGSE-BLADE DWI with SMS (1-min TGSE-BLADE DWI); and to evaluate the diagnostic performance of 1-min TGSE-BLADE DWI for acute or subacute infarction.

## Materials and methods

### Participants

This prospective study was performed in accordance with the Declaration of Helsinki and was approved by Kyoto University Graduate School and Faculty of Medicine, Ethics Committee. Written informed consent was obtained from all participants.

We prospectively enrolled 104 patients with a past history of stroke or symptoms suspicious for acute infarction, or who underwent surgery for a brain tumor within two days, and who underwent SS-EPI DWI, TGSE-BLADE DWI, and T2-weighted imaging (T2WI) between November 2021 and March 2022. The exclusion criteria were as follows: (a) insufficient image quality due to motion artifacts; and (b) unavailability of any of SS-EPI DWI, TGSE-BLADE DWI, or T2WI.

## Image acquisition

MRI was performed using a 3T scanner (MAGNETOM Prisma or MAGNETOM Skyra; Siemens Healthineers, Erlangen, Germany) with a 64-channel head/neck coil or a 32-channel head coil. T2WI of the brain was acquired in addition to the two DWI sequences (SS-EPI DWI and TGSE-BLADE DWI). SS-EPI DWI is a commercially available product that is used routinely in our institute. TGSE-BLADE DWI is a prototype sequence covering the whole brain, and a scan time of 59 s was achieved with a total acceleration factor of 4 (2 × in-plane acceleration and 2 × slice acceleration). The pulse sequence parameters are shown in Table 1.

**Table 1:** Acquisition protocols.

Parameter	SS-EPI DWI	TGSE-BLADE DWI	T2WI
b value (s/mm^2^)	0, 1000	0, 1000	NA
TR (ms)	3900	3300, 3200*	3540
TE (ms)	63, 71*	46, 62*	79
FA (degrees)	NA	120	120
FOV (mm)	220 × 220	220 × 220	220 × 220
Matrix	160 × 160	160 × 160	448 × 448
Slice thickness (mm)	5	5	5
Number of slices	22	22	22
Voxel size (mm^3^)	1.4 × 1.4 × 5.0	1.4 × 1.4 × 5.0	0.5 × 0.5 × 5.0
Bandwidth (Hz/pixel)	1202	520	189
NEX	2	1	1
Parallel imaging (Phase Encoding × Slice Encoding)	GRAPPA 3 × 1	GRAPPA 2 × Slice acceleration 2	GRAPPA 3 × 1
Turbo factor	NA	13	11
EPI factor	128	3	NA
Acquisition time (s)	52	59	51

* Parameters are for MAGNETOM Skyra.

## Image analysis

- Lesion assessment.

Three board-certified neuroradiologists (A.S., S.Ok., and S.Ot. with 16, 16, and 13 years of experience in neuroradiology, respectively) evaluated patients’ images for acute or subacute infarctions, defined as lesions with high signal intensities on b1000 images and without high values on ADC map. High signal intensities on b1000 images were diagnosed as infarction or artifact based on temporal changes and the findings of other MR sequences; e.g., fluid attenuated inversion recovery (FLAIR). In patients who underwent surgery, restricted diffusion due to postoperative changes on images acquired immediately after surgery was diagnosed as acute cerebral infarction or contusion. Any disagreements among the three neuroradiologists were resolved by consensus.

(b) Image quality.

Geometric distortion, susceptibility artifacts, and overall image quality were assessed qualitatively in the b1000 images of all patients using a 4-point Likert scale^7^. In patients who had high signal intensities on b1000 images, lesion conspicuity and diagnostic confidence were qualitatively evaluated in b1000 images using a 4-point Likert scale^7^. In the case of multiple lesions, a comprehensive assessment was performed. The image assessment criteria are listed in Supplementary Table 1. Image quality was evaluated by the same three neuroradiologists who performed lesion assessment. Each reader was blinded to the type of DWI sequence. The majority opinion of the raters was designated as the final score. If the three opinions differed, a resolution was obtained by consensus.

(c) Quantitative analysis.

Distortion was examined quantitatively by measuring the displacement between T2WI and each DWI sequence in three parts of the brain: frontal lobe near frontal sinus, temporal tip, and pons^9^.

Regions-of-interest (ROIs) were placed on high-signal-intensity lesions, centrum semiovale (CSO), and the pons in the b1000 images of each DWI sequence. If multiple lesions were present, the ROI was placed in the slice that contained the greatest area of the largest lesion. In all patients, signal-to-noise ratio (SNR) was calculated as SNR = SI_cso or pons_ / SD_cso or pons_^22^. SI_cso or pons_ and SD_cso or pons_ are the mean and standard deviation of signal intensities of CSO or pons. Contrast-to-noise ratio (CNR) was calculated as CNR = (SI_lesion_ – SI_cso_) / SD_cso_in patients with acute or subacute infarction^22^. SI_lesion_, SI_cso_, and SI_pons_ are the mean signal intensities of lesions of acute or subacute infarction, CSO, and pons, respectively; and SD_cso_is the standard deviation of CSO. The same ROIs were then placed in the ADC maps of each DWI sequence. ROI area was 60–99 mm^2^ in CSO and pons, and 4–69 mm^2^ in lesions. Evaluation of distortion and ROI measurements was performed by a board-certified radiologist (S.Ok.) using ImageJ software version 1.53e (https://imagej.nih.gov/ij/) and was approved by another board-certified radiologist (Y.F. with 25 years of experience in neuroradiology).

## SNR maps

SNR maps were created using SS-EPI DWI and TGSE-BLADE DWI acquired in one healthy volunteer. Each DWI sequence was scanned 10 times, and an SNR map of each DWI was created as the mean map divided by the SD map, using Image Calculator in SPM12 (https://www.fil.ion.ucl.ac.uk/spm/software/).

## Phantom study

A phantom study was performed using Mini Diffusion phantom (CaliberMRI, Inc., Boulder, CO, United States) at MAGNETOM Prisma. The phantom contains three high-performance liquid chromatography water vials (reference) and 10 PVP vials (two each 10%, 20%, 30%, 40%, and 50% PVP). TGSE-BLADE DWI with SMS and TGSE-BLADE DWI without SMS were each scanned 10 times, respectively. The acquisition parameters for both TGSE-BLADE DWI methods were set to a Repetition Time (TR) of 4300 ms, differing from the TR used in the patient study, to enable comparison under the same TR condition. ROIs were placed in the b1000 image and subsequently applied to the ADC maps. The accuracy of each DWI was evaluated by comparison between measured ADC values and theoretical ADC values corresponding to the temperature of the phantom. The SNR map of each DWI was created as the mean map of b1000 images divided by the SD map. The same ROIs were also placed on the SNR map.

### Statistical analysis

Interrater reliability for the image quality scores measured independently by the three radiologists was evaluated using Fleiss’ kappa statistics using RStudio Software version 2022.12.0 (RStudio PBC, Boston, USA)^23^. The calculated κ statistic was interpreted as follows: 0.20 or less, slight agreement; 0.21–0.40, fair agreement; 0.41–0.60, moderate agreement; 0.61–0.80, substantial agreement; and 0.81–1.00, almost perfect agreement.

Lengths of displacement and image quality scores were compared between the two DWI sequences using Wilcoxon signed-rank test because the data distribution was not normal. SNR, CNR, and ADC values were compared between the two DWI sequences using paired t-tests because the data distribution was normal. *p* values less than 0.05 were considered statistically significant. The correlation coefficient was calculated to evaluate correlations of ADC values from the two DWI sequences, and Bland–Altman analysis was also performed. Statistical analyses were performed using MedCalc version 20 (MedCalc Software, Ostend, Belgium).

## Results

### Participants

No participant was excluded from the study. In total, 104 patients were included (mean age, 67.1 ± 16.7; age range, 23–93 years; 64 males, 40 females) (Supplementary Fig. 1). Of 82 patients with a past history of stroke or symptoms suspicious for acute infarction, 37 had high signal intensities indicative of acute or subacute cerebral infarction. Forty-one patients had no high signal intensities on b1000 images, and 4 patients had a high signal intensity on b1000 images that also showed high signal on ADC maps. Twenty-two patients underwent surgery within two days and had high signal intensity lesions indicative of postoperative contusion or acute infarction on b1000 images. Table 2 lists the demographic data of all participants.

**Table 2:** Patient demographics.

Characteristic	Patients with a past history of stroke or symptoms suspicious for acute infarction		Patients who underwent surgery for a brain tumor within two days (*n* = 22)
	Patients without acute or subacute infarction (*n* = 45)	Patients with acute or subacute infarction (*n* = 37)	
Sex (male: female)	27:18	25:12	12:10
Mean age ± SD (years)	68.0 ± 14.9	74.5 ± 11.3	53.0 ± 19.0
Neurological indication for MRI examination	Old cerebral infarction (*n* = 31) Subacute infarction without high signal intensity on b1000 images (*n* = 6) Transient ischemic attack (*n* = 2) Carotid artery stenosis (*n* = 2) Amaurosis fugax (*n* = 1) Old cerebral hemorrhage (*n* = 1) Middle cerebral artery stenosis (*n* = 1) Retinal artery occlusion (*n* = 1)	Acute or subacute infarction Day 1–5 (*n* = 6) Day 6–10 (*n* = 14) Day 11 (*n* = 11) Onset date unknown (*n* = 5) Subarachnoid hemorrhage, post aneurysm coiling (*n* = 1)	Postoperative day 1 of brain tumor Glioma (*n* = 14) Brain metastasis (*n* = 4) Meningioma (*n* = 2) Acoustic schwannoma (*n* = 1) Tuberculoma (*n* = 1)

### Lesion assessment

Ten lesions in 9 patients were diagnosed as acute/subacute infarction or postoperative contusion and were detectable on TGSE-BLADE DWI but not on SS-EPI DWI. These lesions were confirmed to be true lesions, excluding false positive findings clearly, through FLAIR imaging or follow-up DWI on a different day.

**Fig. 1:**
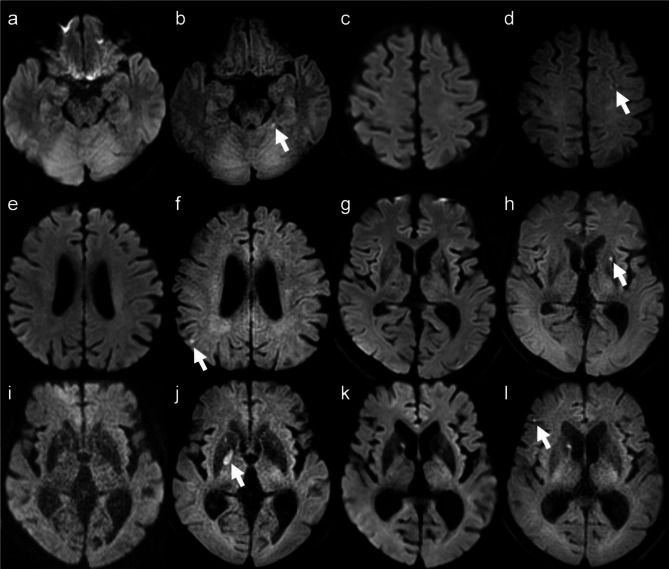
Representative images in patients with acute or subacute infarction. The white arrows indicate infarctions in the cerebellar hemisphere near the cerebellar tentorium (**a**, **b**), left frontal cortex (**c**, **d**), right parietal lobe (**e**, **f**), left putamen (**g**, **h**), right globus pallidus (**i**, **j**), and right frontal cortex (**k**, **l**). SS-EPI DWI (**a**, **c**, **e**, **g**, **i** and **k**); TGSE-BLADE DWI (**b**, **d**, **f**, **h**, **j** and **l**). The lesions are detectable only on TGSE-BLADE DWI but are unclear on SS-EPI DWI due to proximity to cortex, cerebellar tentorium, or hemosiderin deposition in the basal ganglia.

Six of the 10 acute or subacute infarct lesions were in the cerebellar hemisphere near the cerebellar tentorium, frontal cortex, parietal cortex, putamen, and globus pallidus (Fig. 1). All these lesions were very small and located in regions prone to artifacts or near areas of hemosiderin deposition. Additionally, two cases were asymptomatic, while the duration after symptom onset in the remaining four cases ranged from 1 to 4 days, indicating that most lesions were shortly after the onset of infarction.

**Fig. 2:**
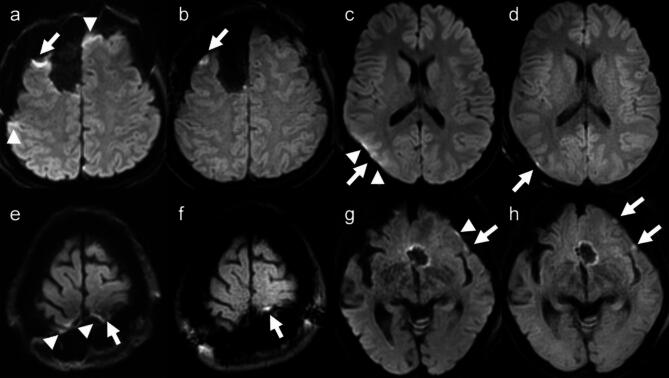
Representative images obtained in patients who underwent surgery for brain tumor show postoperative changes related to acute cerebral infarction or postoperative contusion on postoperative day 1. It is difficult to determine whether the postoperative changes are due to acute cerebral infarction or to postoperative contusion (white arrows) and susceptibility artifact (arrowheads) in SS-EPI DWI (**a**, **c**, **e**, and **g**). Postoperative changes are clearly differentiated as acute cerebral infarction or postoperative contusion on TGSE-BLADE DWI (**b**, **d**, **f**, and **h**).

Four of the 10 acute infarct lesions or postoperative contusions were observed in patients immediately after surgery, and were difficult to find on SS-EPI DWI because of susceptibility artifacts due to air or hemorrhage (Fig. 2). No lesion was detectable on SS-EPI DWI but not on TGSE-BLADE DWI. Lesions visualized only on TGSE-BLADE DWI were verified by pixel-to-pixel comparison in FLAIR images obtained at the same time or in FLAIR images obtained at follow-up MRI.

### Image quality

The kappa values of inter-rater agreement for the image quality scores of geometric distortion, susceptibility artifacts, overall image quality, lesion conspicuity, and diagnostic confidence were 0.67, 0.63, 0.69, 0.49, and 0.54, respectively, showing fair agreement or moderate agreement.

Table 3 lists the image quality scores for each DWI sequence. Scores for geometric distortion, susceptibility artifacts, and overall image quality were lower in SS-EPI DWI than TGSE-BLADE DWI (all *p* < .001). Scores for lesion conspicuity and diagnostic confidence were lower in SS-EPI DWI than TGSE-BLADE DWI in patients with acute infarction and in patients immediately after surgery (*p* ≤ .001 and *p* < .001, respectively).

**Table 3:** Results of image quality for SS-EPI DWI and TGSE-BLADE DWI.

The scores of geometric distortion, susceptibility artifacts and overall image quality (*n* = 104)			
	SS-EPI DWI	1-min TGSE-BLADE DWI	*p* value
Geometric distortion	3.0 (3.0–3.0)	4.0 (4.0–4.0)	< 0.001
Susceptibility artifacts	3.0 (3.0–3.0)	4.0 (4.0–4.0)	< 0.001
Overall image quality	3.0 (3.0–3.0)	4.0 (4.0–4.0)	< 0.001
The scores of lesion conspicuity and diagnostic confidence in patients with acute or subacute infarctions (*n* = 37)			
	SS-EPI DWI	1-min TGSE-BLADE DWI	*p* value
Lesion conspicuity	4.0 (3.1–4.0)	4.0 (4.0–4.0)	0.001
Diagnostic confidence	4.0 (3.0–4.0)	4.0 (4.0–4.0)	< 0.001
The scores of lesion conspicuity and diagnostic confidence in patients who have undergone surgery for a brain tumor within a few days (*n* = 22)			
	SS-EPI DWI	1-min TGSE-BLADE DWI	*p* value
Lesion conspicuity	3.0 (3.0–3.0)	4.0 (4.0–4.0)	< 0.001
Diagnostic confidence	3.0 (3.0–3.0)	4.0 (4.0–4.0)	< 0.001

Note – Data are presented as the median (interquartile range).

### Quantitative analysis

**Fig. 3:**
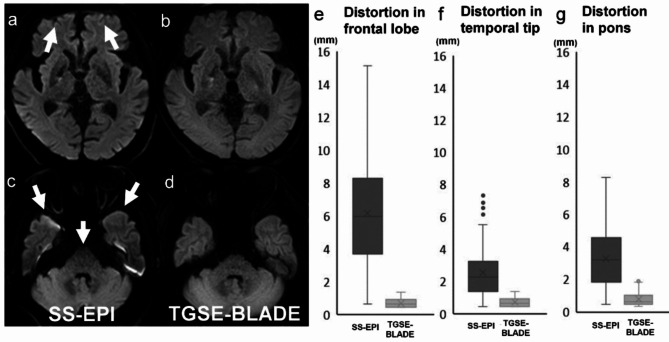
A 74-year-old male with acute infarctions in right basal ganglia and corona radiata. Values of distortion seen on SS-EPI DWI (**a**, **c**) and TGSE-BLADE DWI (**b**, **d**) are shown in boxplots (**e**–**g**) for the regions of frontal lobe near frontal sinus (**e**), temporal tip (**f**), and pons (**g**). In all three regions, distortion was less pronounced in TGSE-BLADE DWI than SS-EPI DWI.

Example images and the measured distortion values are shown in Fig. 3. Distortion values were significantly higher in SS-EPI DWI than TGSE-BLADE DWI in frontal lobe, temporal tip, and pons (*p* < .001).

**Fig. 4:**
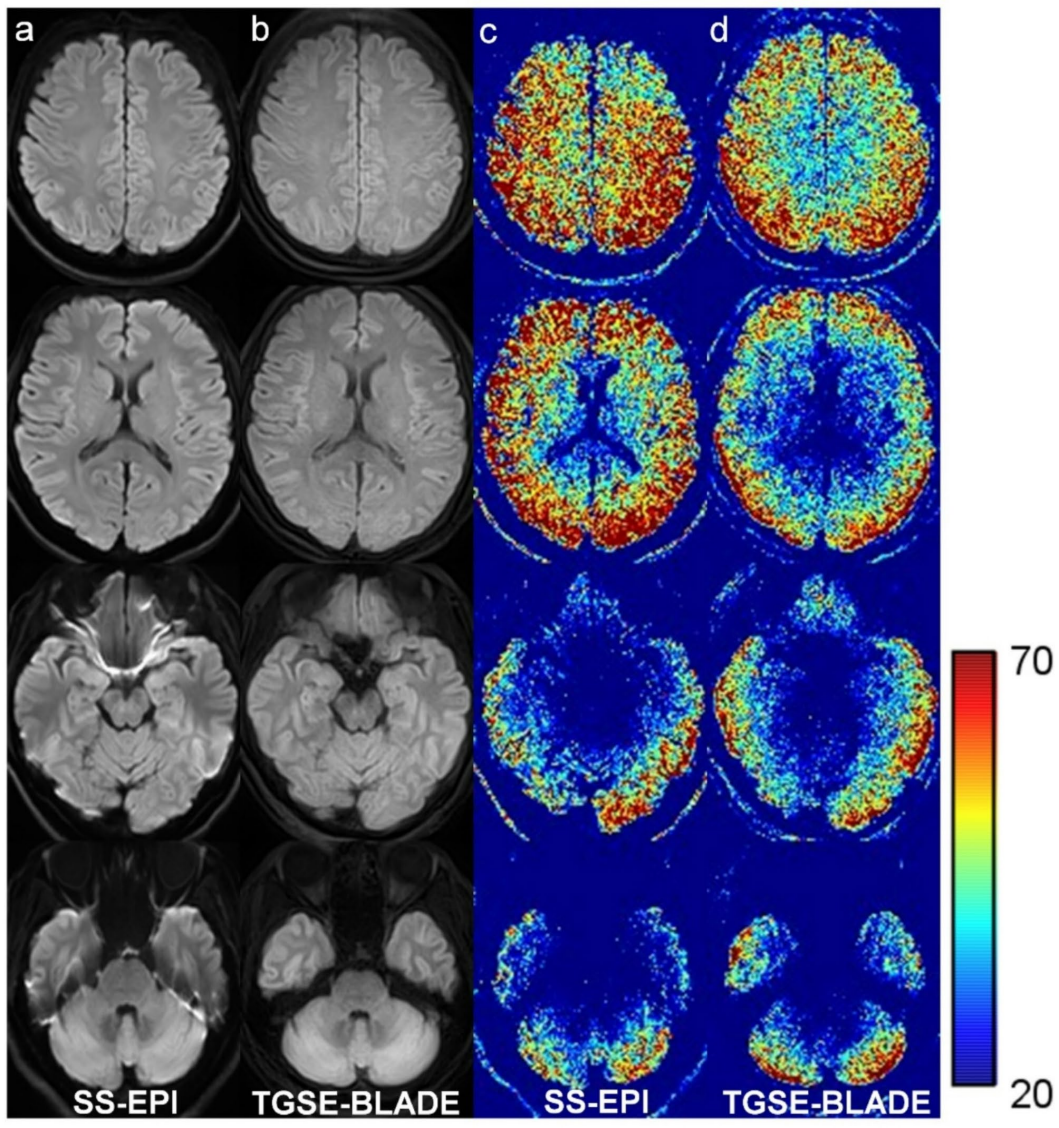
Representative b1000 images (**a**, **b**) and SNR maps (**c**, **d**) of SS-EPI DWI (**a**, **c**) and TGSE-BLADE DWI (**b**, **d**) are shown in a healthy volunteer.

Mean SNR in CSO was significantly higher in SS-EPI DWI (26.3 ± 7.0) than TGSE-BLADE DWI (22.0 ± 5.5) (*p* < .001), but showed no significant difference in pons (SS-EPI DWI, 9.7 ± 3.0; TGSE-BLADE DWI, 9.5 ± 1.8) (*p* = .40). Mean SNR values were higher at the periphery and lower at the center of the brain in the SNR maps for both DWI sequences due to the characteristics of the 32-channel phased array coil (Fig. 4). Mean SNR in CSO was higher in SS-EPI DWI than TGSE-BLADE DWI; however, SNR in temporal lobe was higher in TGSE-BLADE DWI, probably because this sequence is less prone to susceptibility artifacts. Mean CNR was significantly higher in SS-EPI DWI (20.5 ± 12.1) than TGSE-BLADE DWI (15.5 ± 11.1) (*p* < .001).

Mean ADC values for each DWI are shown in Table 4. There was no significant difference in ADC values in CSO or pons. In lesions, mean ADC values were significantly lower in SS-EPI DWI than TGSE-BLADE DWI (*p* = .004). There was a linear correlation between SS-EPI DWI and TGSE-BLADE DWI for ADC values in lesions (*r* = .80) (Supplementary Fig. 2a). Bland–Altman analysis of the ADC measurements of SS-EPI DWI and TGSE-BLADE DWI revealed that most data were distributed between ± 1.96 SD (Supplementary Fig. 2b).

**Table 4:** ADC values in centrum semiovale, pons, and lesions for SS-EPI DWI and TGSE-BLADE DWI.

	SS-EPI DWI	TGSE-BLADE DWI	*p* value
CSO	769.6 ± 81.8	764.8 ± 67.8	0.37
Pons	761.2 ± 53.2	769.0 ± 98.1	0.42
Lesions	592.9 ± 146.2	627.2 ± 124.2	0.004

Note – Data are presented as the mean ± SD (mm^2^/s). CSO, centrum semiovale.

### Phantom study

The SNR maps are presented in Supplementary Fig. 3, and the Measured ADC, theoretical ADC, and SNR values are summarized in Supplementary Table 2. The temperature was 24.0℃. The mean SNR value showed minimal difference between scans performed with and without SMS. Additionally, the ADC values obtained from TGSE-BLADE DWI without SMS were closer to the theoretical values compared to those obtained with SMS.

## Discussion

The major findings of the present study are that some acute infarctions were detectable only by TGSE-BLADE DWI whereas no lesions were detectable only by SS-EPI DWI, and that scores for geometric distortion, susceptibility artifacts, overall image quality, lesion conspicuity, and diagnostic confidence were higher for TGSE-BLADE DWI. Taken together, these imaging image characteristics indicate the potential utility of TGSE-BLADE DWI with SMS for diagnosis of acute infarction. In previous studies with TGSE-BLADE DWI, scan time was consistently 4 to 5 min^4–9^. The present study reports the first attempt to significantly reduce acquisition time to approximately 1 min. The ability to scan images within this shortened timeframe, coupled with enhanced diagnostic capabilities for acute cerebral infarction compared to SS-EPI DWI, renders it highly valuable for routine clinical application.

Lesions that could not be identified after surgery as acute infarction on SS-EPI DWI were located near the cerebellar tentorium, cortex, hemorrhage, or pneumocephalus. A previous study has reported sensitivity of 81.1% and a false-negative rate of 5.6% for detecting infratentorial infarctions using 5 mm SS-EPI DWI, and lesions in false-negative cases were small^24^. Another study noted that most patients with false-negative lesions had infratentorial infarction or transient ischemic attack^25^. To mitigate false negatives, several reports have suggested that incorporating coronal sections or thin slice DWI can enhance diagnostic capabilities with SS-EPI DWI^26,27^. However, it might be possible to diagnose acute cerebral infarctions prone to false negatives using TGSE-BLADE DWI alone, and achieve diagnostic accuracy similar to that of additional imaging (such as coronal sections or thin slices) without acquiring additional scans.

Median scores for geometric distortion and susceptibility artifacts were 3.0 for SS-EPI DWI and 4.0 for TGSE-BLADE DWI. Distortion was also quantitatively less near the air-bone interfaces (e.g., frontal lobe, temporal tip, and pons) in TGSE-BLADE-DWI with SMS. These findings align with those of a prior study that used TGSE-BLADE DWI without acceleration technique^9^. Their scores for lesion conspicuity and diagnostic confidence in patients with acute or subacute infarction were lower in SS-EPI DWI than TGSE-BLADE DWI; however, median score was 4.0 for each sequence. In contrast, median score in post-surgery patients was 3.0 for SS-EPI DWI and 4.0 for TGSE-BLADE DWI. We consider that there is greater susceptibility postoperatively to artifacts due to air or hemorrhage, in which case TGSE-BLADE DWI is more beneficial.

SNR values were lower for TGSE-BLADE DWI than SS-EPI DWI in CSO. In SNR maps for temporal lobe, however, values were higher for TGSE-BLADE DWI than for SS-EPI DWI. TGSE-BLADE DWI showed less SNR degradation in areas prone to distortion, such as near air–bone interfaces, whereas SS-EPI DWI demonstrated superior SNR in other regions, primarily because only half of the signals are used in this sequence due to the non-CPMG (Carr-Purcell-Meiboom-Gill) problem. Another reason for the lower SNR values is that positioning the gradient echo with T2* decay effects at the center of k-space diminishes the image quality of TGSE-BLADE DWI^5^. Despite these disadvantages of 1-min TGSE-BLADE DWI, its ability to detect lesions located near susceptibility artifacts is a strong advantage.

There was no significant difference between the sequences in terms of ADC values in CSO or pons. In lesions, however, ADC values were significantly higher for TGSE-BLADE DWI than SS-EPI DWI, consistent with the findings of a previous study^6^. This discrepancy might have been due to the substantial differences in SNR and T1 values between normal tissue and lesions^28^, but the cause remains unclear because no study has investigated cerebral infarction using TGSE-BLADE DWI. Moreover, differences in diffusion time or echo time (TE) between sequences might have contributed to the observed ADC differences, as a previous report suggests these parameters can influence ADC measurements^29^. These differences might also have contributed to the lower CNR observed in TGSE-BLADE DWI. Whereas there was a strong correlation in ADC values for lesions between SS-EPI DWI and TGSE-BLADE DWI. Therefore, we consider that there should be few issues in clinical diagnosis.

There are several limitations in this study. Firstly, the sample size was small. A larger sample size might facilitate a more comprehensive investigation of lesions that could not be visualized by SS-EPI DWI. However, we prospectively enrolled over 100 patients, and believe that the number of cases was sufficient to demonstrate the utility of TGSE-BLADE DWI. Second, subacute infarct was diagnosed most commonly, and there were relatively few hyperacute infarcts. Due to the prospective nature of the study in which two types of DWI were acquired, it was challenging to perform these imaging examinations in patients with hyperacute stroke who require urgent treatment decisions. A previous study reported that some lesions were not depicted on SS-EPI DWI in the hyperacute stage^30^, indicating the need for further investigation in the future. Third, SMS imaging was not applied for SS-EPI DWI because we compared TGSE-BLADE DWI with SS-EPI DWI acquired with the protocol used at our institution. Although it is feasible to implement SMS for SS-EPI DWI, there is limited advantage because the shorter TR used has the effect of reducing SNR. Finally, in making the score judgments, the neuroradiologists noted that it was easy to distinguish the SS-EPI DWI and TGSE-BLADE DWI sequences based on the presence or absence of signal pile-up and geometric distortion.

## Conclusion

Compared with SS-EPI DWI, one-minute TGSE-BLADE DWI has better image quality in terms of distortion and artifacts, higher diagnostic performance for identifying acute infarctions, and its acquisition time is similar to that of SS-EPI DWI. One-minute TGSE-BLADE DWI is therefore clinically acceptable and shows promise as a diagnostic tool for identifying acute infarctions in acute stroke patients and postoperative patients.

## Electronic supplementary material

Below is the link to the electronic supplementary material.

Supplementary Material 1

## Data Availability

The datasets used and/or analyzed during the current study are available from the corresponding author on reasonable request.

## Declarations

### Yuta Urushibata MSci

Yuta Urushibata is an employee of Siemens Healthcare K. K., Japan.
