# Supplementary material for: Comparison of SS-EPI DWI and one-minute TGSE-BLADE DWI for diagnosis of acute infarction
Source: Sci Rep. 2025 Feb 22;15:6512. doi: 10.1038/s41598-025-90413-5 (PMC11846894; doi:10.1038/s41598-025-90413-5)
Supplement: Supplementary file 1 — Supplementary Material 1 [file 41598_2025_90413_MOESM1_ESM.docx]

**Supplementary Table 1**

Image assessment criteria

|  | Geometric distortion | Susceptibility artifacts | Overall image quality | Lesion conspicuity (contrast between lesion and surrounding background) | Diagnostic confidence for acute infarction |
| --- | --- | --- | --- | --- | --- |
| 1 | Severe distortion | Severe artifacts | Poor, insufficient for diagnosis | Unable to evaluate | Vague (10–39%) |
| 2 | Moderate distortion | Major artifacts | Fair, adequate for diagnosis | Acceptable for visualization | Likely (40–69%) |
| 3 | Mild distortion | Only minor artifacts | Good for diagnosis | Obvious visibility | High (70–89%) |
| 4 | No distortion | No artifacts | Excellent for diagnosis | Excellent for visualization | Definite (90–100%) |

**Supplementary Table 2**

Theoretical and measured ADC values (mean ± SD) [10^-6^ mm^2^/sec] and SNR (mean) in a phantom study

|  |  | **ADC** | | | **SNR** | |
| --- | --- | --- | --- | --- | --- | --- |
|  |  | **Theoretical Value** | **TGSE-BLADE without SMS** | **TGSE-BLADE with SMS** | **TGSE-BLADE without SMS** | **TGSE-BLADE with SMS** |
| ROI 1 | Water | 2225 | 2160.2 ± 3.0 | 2102.9 ± 2.1 | 99.8 | 96.9 |
| ROI 2 | Water | 2225 | 2165.9 ± 2.1 | 2139.2 ± 1.5 | 98.9 | 86.2 |
| ROI 3 | Water | 2225 | 2225.0 ± 2.2 | 2174.4 ± 1.4 | 88.1 | 70.8 |
| ROI 4 | PVP10 | 1727 | 1735.3 ± 1.4 | 1754.7 ± 1.6 | 115.5 | 100.5 |
| ROI 5 | PVP10 | 1727 | 1704.9 ± 2.0 | 1715.3 ± 1.0 | 134.2 | 125.5 |
| ROI 6 | PVP20 | 1313 | 1312.7 ± 1.8 | 1356.8 ± 1.2 | 203.4 | 206.4 |
| ROI 7 | PVP20 | 1313 | 1338.0 ± 1.9 | 1358.8 ± 1.0 | 270.8 | 237.8 |
| ROI 8 | PVP30 | 960 | 958.5 ± 1.8 | 1015.1 ± 0.6 | 289.2 | 321.4 |
| ROI 9 | PVP30 | 960 | 976.2 ± 1.8 | 1016.9 ± 0.7 | 346.9 | 297.4 |
| ROI 10 | PVP40 | 585 | 594.2± 1.1 | 685.6 ± 0.4 | 374.1 | 402.8 |
| ROI 11 | PVP40 | 585 | 606.7 ± 0.9 | 673.1 ± 0.5 | 412.6 | 376.5 |
| ROI 12 | PVP50 | 356 | 361.1 ± 0.7 | 476.5 ± 0.5 | 383.9 | 457.4 |
| ROI 13 | PVP50 | 356 | 389.1 ± 0.8 | 432.5 ± 0.5 | 511.0 | 473.8 |

**Supplementary Figure 1**

**
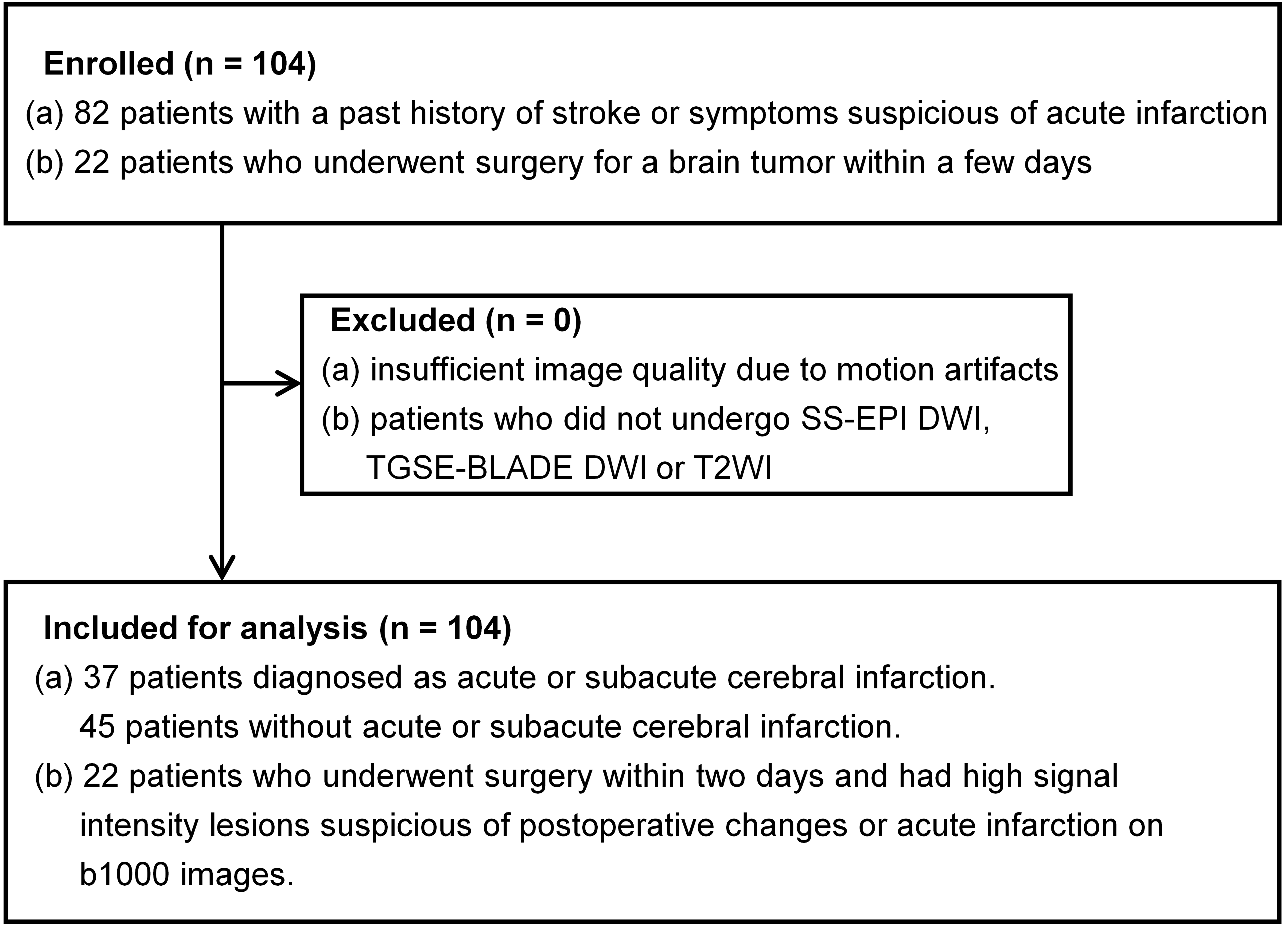
**

Flowchart of study enrollment.


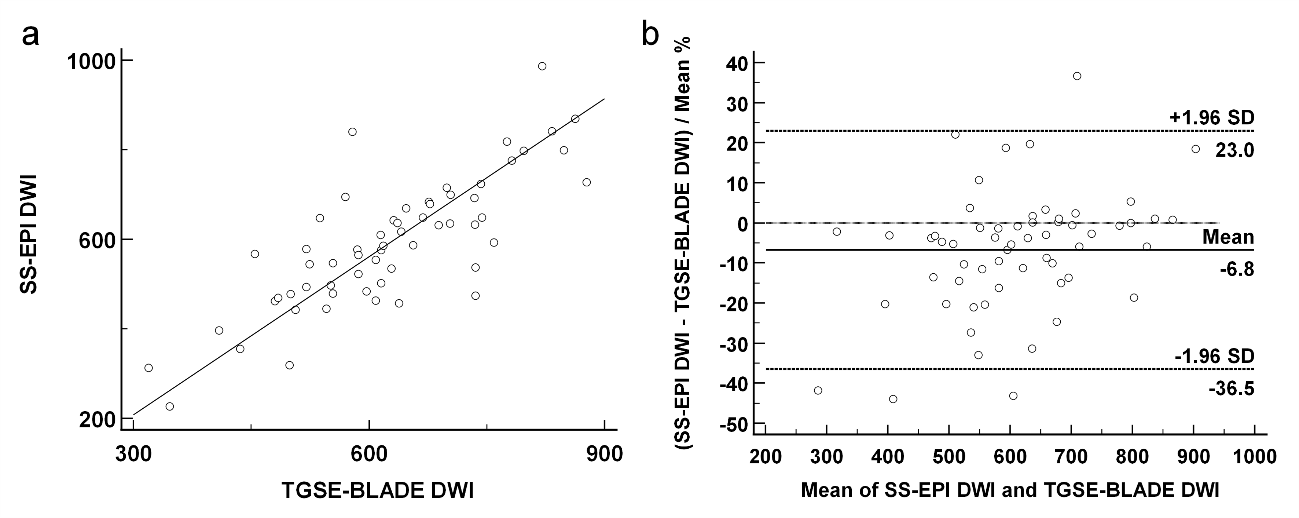
**Supplementary Figure 2**

(a) Scatter plots of ADC values in lesions show a linear correlation between SS-EPI DWI and TGSE-BLADE DWI. The correlation was very strong (r=0.80). (b) Bland–Altman analysis performed between the ADC values of SS-EPI DWI and TGSE-BLADE DWI showed that most data were distributed between ±1.96 SD.

**
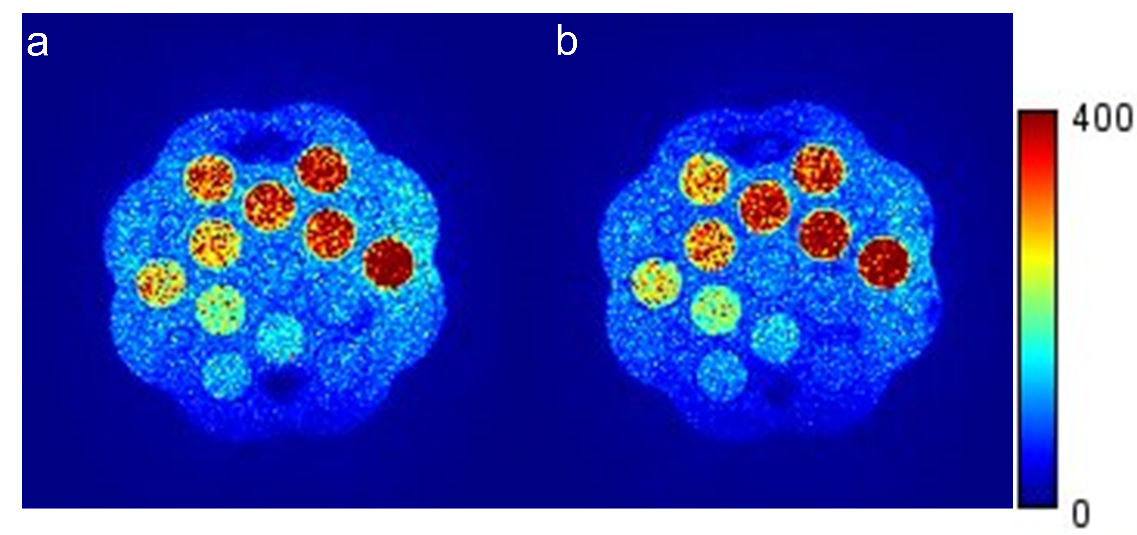
Supplementary Figure 3**

The SNR maps of TGSE-BLADE DWI without SMS (a) and TGSE-BLADE DWI with SMS (b) in a phantom study.
